# Evaluation of Maternal Toxicity in Rats Exposed to 1,3-Dichloro-2-propanol during Pregnancy

**DOI:** 10.5487/TR.2008.24.4.307

**Published:** 2008-12-01

**Authors:** Jong-Chan Lee, In-Sik Shin, Gang-Hyeon Kim, Na-Hyeong Park, Chang-Jong Moon, Chun-Sik Bae, Sung-Soo Kang, Sung-Ho Kim, Dong-Ho Shin, Jong-Choon Kim

**Affiliations:** Animal Medical Center, College of Veterinary Medicine, Chonnam National University, Gwangju, 500-757 Korea

**Keywords:** 1,3-Dichloro-2-propanol, Pregnancy, Maternal toxicity, Serum biochemistry, Rats

## Abstract

The present study was carried out to investigate the potential adverse effects of 1,3-dichloro-2-propanol on pregnant dams after maternal exposure during the gestational days (GD) 6 through 19 in Sprague-Dawley rats. The tested chemical was administered orally to pregnant rats at dose levels of 0, 10, 30, or 90 mg/kg/day. During the test period, clinical signs, mortality, body weights, food consumption, serum biochemistry, gross findings, organ weights, and Caesarean section findings were examined. In the 90 mg/kg group, decreases in the body weight gain and food consumption, and increases in the weights of liver and adrenal glands were observed. Serum biochemical investigations revealed increases in aspartate aminotransferase (AST), alanine aminotransferase (ALT), cholesterol (CHO), triglyceride (TG), alkaline phosphatase (ALP), and bilirubin (BIL) and decreases in glucose (GLU), albumin (ALB) and total protein (TP). In the 30 mg/kg group, a decrease in the food consumption and an increase in the liver weight were observed. Serum biochemical investigation also showed increases in CHO and TG and a decrease in glucose. Since there were no signs of maternal toxicity in the 10 mg/kg group, it is considered to be the no-observed-adverse-effect level (NOAEL) of 1,3-dichloro-2-propanol. It is concluded that successive oral administration of 1,3-dichloro-2-propanol to pregnant rats for 14 days may cause significant toxicities in body weight and liver at a dose rate ≥ 30 mg/kg/day.

## INTRODUCTION

Volatile organic compounds (VOCs) have high vapor pressures and are easily vaporized at ambient temperature and pressure. Most hydrocarbons, including nitrogenous, chlorinated, and sulfurated organics, can be categorized as VOCs. These compounds are usually utilized in industries that manufacture and/or use organic solvents, e.g. petrochemical, pulp or coating industries.

Dichloropropanols are a family of chlorinated semi-VOCs that are used in industries such as hard resin production, water chlorination and paper fabrication (Garle *et al.*, 1999; Hammond and Fry, 1999). 1,3-Dichloro-2-propanol (1,3-DCP) is used in high volume as an intermediate in the production of epichlorohydrin, the monomer which is widely used in the production of epoxy resin. Dehydration of 1,3-DCP with phosphoryl chloride forms 1,3-dichloropropene, a soil fumigant. Chlorination of 1,3-DCP with phosphorus pentachloride yields 1,2,3-trichloropropane. Hydrolysis of dichlorohydrins has been used in the production of synthetic glycerol. Therefore, workers may be exposed to 1,3-DCP during the manufacture and the use of these chemicals. Exposure to 1,3-DCP may also occur from ingestion of food to which hydrochloric acid-hydrolyzed vegetable protein has been added or drinking water in which epichlorohydrin polyamine polyelectrolytes are used as flocculents and coagulants for water purification (Nyman *et al.*, 2003).

The most common adverse effects associated with 1,3-DCP in humans are hepatotoxicity, irritation of the mucous membranes, eyes and skin, as well as nausea and vomiting (Shiozaki *et al.*, 1994). The potential toxicity of 1,3-DCP has been studied extensively over the past several decades using both short- and long-term animal tests. The acute oral $LD_{50}$ of 1,3-DCP was ranged from 110 to 400 mg/kg in rats and from 25 to 125 mg/kg in mice, depending upon the purity of 1,3-

DCP and the rodent sex and strain used (RTECS, 2000; HSDB, 2002). The major adverse effects observed in humans exposed to 1,3-DCP were well demonstrated in experimental animal studies (Jersey *et al.*, 1991; Hammond *et al.*, 1996; Katoh *et al.*, 1998; Lym *et al.*, 2003). It was also reported that 1,3-DCP was mutagenic and genotoxic in various bacterial and mammalian test systems *in vitro* (Zeiger *et al.*, 1988; Hahn *et al.*, 1991; Ohkubo *et al.*, 1995) and carcinogenic in both rats and mice (Hercules Inc., unpublished results; Eder and Weinfurtner, 1994) and an in vitro system (Piasecki *et al.*, 1990). It has been reported that the toxic mechanisms of 1,3-DCP are lipid peroxidation (Katoh *et al.*, 1998; Kuroda *et al.*, 2002), glutathione depletion (Katoh *et al.*, 1998; Garle *et al.*, 1999; Fry *et al.*, 1999) and disruption of the mitochondrial membrane potential (Hammond et al., 1996). The metabolite 1,3-dichloroacetone (1,3-DCA) was identified to be responsible for necrosis of the liver and other disorders (Hahn *et al.*, 1991; Shiozaki *et al.*, 1994).

The potential risk caused by 1,3-DCP in humans has recently increased due to its increased production and widespread use, which may result in severe health impacts caused by indoor contamination in the home and workplace. However, the potential adverse effects of 1,3-DCP on pregnant dams have not been studied yet. Therefore, the present study was carried out to investigate the potential effect of 1,3-DCP on pregnant dams of Sprague-Dawley rats when administered from days 6 through 19 of gestation.

## MATERIALS AND METHODS

***Animal husbandry and maintenance.*** Male and nulliparous female Sprague-Dawley rats aged 10 weeks were obtained from a specific pathogen free colony at Bio Genomics Inc. (Seoul, Republic of Korea). The animals were used after one week of quarantine and acclimatization. Rats were housed in a room maintained at a temperature of $23 \pm 3^oC$ and a relative humidity of $50 \pm 10\%$ with artificial lighting from 08:00 to 20:00 and with 13 to 18 air changes per hour. Only healthy animals were assigned to the study. For mating, two females were placed into the cage of each male overnight. Successful mating was ascertained by the presence of sperm in a vaginal smear, and the following first 24 h was designated as day 0 of gestation (GD 0). Mated females were housed singly in clear polycarbonate cages with stainless steel wire lids and were allowed sterilized tap water and commercial rodent chow (PMI Nutrition International, IN, USA) *ad libitum*. The Institutional Animal Care and Use Committee of Chonnam National University approved the protocols for the animal study, and the animals were cared in accordance with the Guidelines for Animal Experiments of Chonnam National University.

***Test chemical and treatment.*** 1,3-DCP was purchased from Acros Orgarnics (Fisher Scientific Korea city, Republic of Korea). The tested chemical was freshly prepared in sterilized distilled water daily before the treatment. The rats were administered the mixture orally that was calculated in advance based on the most recently recorded body weight of the individual animal. 1,3-DCP was administered to each pregnant rat from GD 6 through 19 with a dose volume of 5 ml/kg body weight. The vehicle control rats received an equivalent volume of distilled water alone.

***Experimental group.*** Healthy female rats were assigned randomly to four experimental groups: three treatment groups of 1,3-DCP receiving 10, 30, or 90 mg/kg/day and a vehicle control group ($n = 10$ inseminated females per group).

***Observation of dams.*** All pregnant females were observed daily throughout gestation for mortality, morbidity, general appearance and behavior. Maternal body weights were measured on GD 0, 6, 9, 12, 15, and 20 and individual food consumption was determined on GD 0, 6, 9, 12, 15, and 19. At scheduled termination (GD 20), all pregnant females were euthanized by ether inhalation and subjected to external and internal macroscopic examination. The absolute and relative (organ-to-body weight ratios) weights of the following organs were measured: lung, adrenal gland, liver, spleen, kidney, thymus, heart, and ovary.

***Serum biochemistry.*** Blood samples were drawn from the posterior vena cava using a syringe with 24-gauge needle under ether anesthesia. The samples were centrifuged at 3000 rpm for 10 min within 1 h after collection. The sera were stored at $-80^oC$ in a freezer prior to analysis. The following serum biochemistry parameters were evaluated using an autoanalyzer (Shimadzu CL-7200, Shimadzu Co.): aspartate aminotransferase (AST), alanine aminotransferase (ALT), cholesterol (CHO), triglyceride (TG), blood urea nitrogen (BUN), alkaline phosphatase (ALP), creatinine (CRE), glucose (GLU), calcium (CA), bilirubin (BIL), albumin (ALB), and total protein (TP).

***Caesarean section.*** The ovaries and uterus of each female were removed and examined for the number of corpora lutea and the status of all implantation sites, i.e., live and dead fetuses, early and late resorptions and total implantations. Resorption was classified as "early" when only placental tissue was visible and "late" when placental and embryonic tissue were visible at caesarean section. All live fetuses were weighed and sexed individually.

***Statistical analyses.*** The results are expressed as mean ± SD. Quantitative continuous data such as the maternal body weight, food consumption, fetal body weight, placental weight, and serum biochemical values were subjected to a one-way analysis of the variance (ANOVA), and a Scheffe multiple comparison test was carried out when the differences were significant (Scheffe, 1953). The numbers of corpora lutea, total implantations, live and dead fetuses were statistically evaluated using the Kruskal-Wallis nonparametric ANOVA (Kruskal and Wallis, 1952), followed by the Mann-Whitney U test when appropriate. The gender ratio of live fetuses was compared using the chi-square test and Fisher's exact probability test. The difference was considered statistically significant when $p \leq 0.05$.

## RESULTS

***Clinical signs.*** Pregnant dams in the 30- and/or 90-mg/kg groups showed treatment-related clinical signs such as piloerection, abnormal gait, decreased locomotor activity, a reddish tear from the eyes, loss of fur, and nasal discharge, which were all dose dependent in both the incidence and severity (Table 1).

***Body weight changes and food consumption.*** As shown in Table 2, the body weight gain of pregnant rats was significantly decreased in 90 mg/kg group compared with the control group on GD 9. Additionally, corrected body weight was significantly decreased in the 90 mg/kg group compared with the control group. As presented in Table 3, the amount of food consumed was significantly lower on GD 6 in the 30 mg/kg group and GD 6 and 9 in the 90 mg/kg group when compared with the control group in a dose-dependent manner.

**Table 1.** Clinical findings of pregnant rats treated with 1,3-dichloro-2-propanol during gestational days 6 through 19

| Items | 1,3-Dichloro-2-propanol (mg/kg/day) | | | |
|---|---|---|---|---|
| | 0 | 10 | 30 | 90 |
| No. of mated females | 10 | 10 | 10 | 10 |
| No. of pregnant females | 8 | 8 | 9 | 10 |
| No. of dams with clinical signs[a] | 2 | 1 | 5 | 8 |
| Piloerection | 0 | 0 | 1 | 3 |
| Abnormal gait | 0 | 0 | 0 | 3 |
| Decreased locomotor activity | 0 | 0 | 3 | 5 |
| Reddish tear | 1 | 0 | 0 | 2 |
| Loss of fur | 1 | 1 | 2 | 3 |
| Nasal hemorrhage | 0 | 0 | 0 | 2 |

[a]A single dam may be represented more than once in listing individual signs.

***Gross findings.*** At the scheduled necropsy, there were no treatment-related gross findings in any of the treated animals (data not shown).

***Organ weights.*** The absolute weights of liver in the 30 and 90 mg/kg groups were significantly increased in a dose-dependent manner compared with that of the control group (Table 4). As shown in Table 5, the relative weights of adrenal glands, liver and kidneys in the

**Table 2.** Body weight of the pregnant rats treated with 1,3-dichloro-2-propanol during gestational days 6 through 19

| Items | 1,3-Dichloro-2-propanol (mg/kg/day) | | | |
|---|---|---|---|---|
| | 0 | 10 | 30 | 90 |
| Number of pregnant rats | 8 | 8 | 9 | 10 |
| Gestational day 0 | 239.6 ± 11.13[a] | 238.7 ± 15.79 | 239.0 ± 13.36 | 240.1 ± 6.14 |
| Gestational day 6 | 265.8 ± 14.68 | 268.2 ± 18.62 | 265.9 ± 14.71 | 268.2 ± 6.01 |
| Gestational day 9 | 277.9 ± 14.73 | 270.5 ± 27.35 | 263.8 ± 12.73 | 247.4 ± 15.05** |
| Gestational day 12 | 287.5 ± 17.60 | 272.2 ± 33.37 | 274.2 ± 16.77 | 267.3 ± 8.94 |
| Gestational day 15 | 302.8 ± 18.91 | 297.4 ± 19.74 | 290.9 ± 14.69 | 285.7 ± 10.26 |
| Gestational day 20 | 367.8 ± 27.78 | 358.3 ± 20.94 | 361.9 ± 20.13 | 354.6 ± 22.88 |
| Body weight gain | | | | |
| Pre-treatment period | 28.4 ± 4.31 | 29.7 ± 8.55 | 26.9 ± 4.46 | 28.2 ± 5.88 |
| Treatment period | 98.8 ± 24.57 | 86.9 ± 9.31 | 96.0 ± 19.03 | 86.4 ± 20.83 |
| Corrected body weight[b] | 302.6 ± 19.48 | 288.1 ± 14.39 | 284.2 ± 13.99 | 274.4 ± 13.56** |
| Gravid uterine weight | 65.2 ± 21.24 | 70.2 ± 15.28 | 77.7 ± 11.62 | 80.2 ± 12.48 |

[a]Values are presented as means ± SD (g).
[b]Corrected body weight = Body weight on gestational day 20-gravid uterine weight.
**Significant difference at $p < 0.01$ level when compared with the control group.

**Table 3.** Food consumption of the pregnant rats treated with 1,3-dichloro-2-propanol during gestational days 6 through 19

| Items | 1,3-Dichloro-2-propanol (mg/kg/day) | | | |
|---|---|---|---|---|
| | 0 | 10 | 30 | 90 |
| Number of pregnant rats | 8 | 8 | 9 | 10 |
| Gestational day 0 | 17.8 ± 3.86[a] | 19.6 ± 2.41 | 18.2 ± 2.18 | 18.4 ± 3.12 |
| Gestational day 6 | 18.3 ± 6.65 | 15.1 ± 5.62 | 9.8 ± 3.33** | 7.2 ± 3.51** |
| Gestational day 9 | 21.9 ± 2.29 | 18.6 ± 4.06 | 19.0 ± 4.21 | 16.1 ± 3.90** |
| Gestational day 12 | 21.1 ± 3.49 | 19.4 ± 4.66 | 18.3 ± 7.08 | 16.7 ± 5.54 |
| Gestational day 15 | 20.3 ± 4.22 | 19.3 ± 3.96 | 20.1 ± 4.46 | 18.6 ± 7.14 |
| Gestational day 19 | 22.4 ± 2.66 | 22.2 ± 2.76 | 22.9 ± 4.98 | 22.4 ± 3.18 |

[a]Values are presented as means ± SD (g).
**Significant difference at $p < 0.01$ level when compared with the control group.

**Table 4.** Absolute organ weights of pregnant rats treated with 1,3-dichloro-2-propanol during gestational days 6 through 19

| Items | 1,3-Dichloro-2-propanol (mg/kg/day) | | | |
|---|---|---|---|---|
| | 0 | 10 | 30 | 90 |
| Number of pregnant rats | 8 | 8 | 9 | 10 |
| Lung (g) | 1.23 ± 0.082[a] | 1.17 ± 0.116 | 1.27 ± 0.139 | 1.32 ± 0.210 |
| Adrenal glands (g) | 0.05 ± 0.018 | 0.06 ± 0.012 | 0.06 ± 0.018 | 0.07 ± 0.016 |
| Liver (g) | 13.31 ± 1.163 | 14.43 ± 1.002 | 17.08 ± 2.166** | 18.69 ± 1.751** |
| Spleen (g) | 0.70 ± 0.170 | 0.59 ± 0.077 | 0.66 ± 0.106 | 0.65 ± 0.097 |
| Kidneys (g) | 1.82 ± 0.223 | 1.83 ± 0.151 | 1.98 ± 0.201 | 2.01 ± 0.109 |
| Thymus (g) | 0.23 ± 0.041 | 0.24 ± 0.075 | 0.20 ± 0.075 | 0.20 ± 0.055 |
| Heart (g) | 0.95 ± 0.111 | 0.90 ± 0.110 | 0.90 ± 0.106 | 0.86 ± 0.071 |
| Ovaries (g) | 0.10 ± 0.017 | 0.09 ± 0.022 | 0.09 ± 0.013 | 0.10 ± 0.016 |

[a]Values are presented as means ± SD.
**Significant difference at $p < 0.01$ level when compared with the control group.

**Table 5.** Relative organ weights of pregnant rats treated with 1,3-dichloro-2-propanol during gestational days 6 through 19

| Items | 1,3-Dichloro-2-propanol (mg/kg/day) | | | |
|---|---|---|---|---|
| | 0 | 10 | 30 | 90 |
| Number of pregnant rats | 8 | 8 | 9 | 10 |
| Body weight (g) | 367.8 ± 27.78[a] | 358.3 ± 20.94 | 361.9 ± 20.13 | 354.6 ± 22.88 |
| Lung (%) | 0.34 ± 0.037 | 0.33 ± 0.031 | 0.35 ± 0.039 | 0.37 ± 0.061 |
| Adrenal glands (%) | 0.01 ± 0.005 | 0.02 ± 0.003 | 0.02 ± 0.006 | 0.02 ± 0.004* |
| Liver (%) | 3.62 ± 0.243 | 4.04 ± 0.325 | 4.71 ± 0.392** | 5.28 ± 0.440** |
| Spleen (%) | 0.19 ± 0.043 | 0.16 ± 0.020 | 0.18 ± 0.027 | 0.19 ± 0.033 |
| Kidneys (%) | 0.50 ± 0.070 | 0.51 ± 0.055 | 0.55 ± 0.041 | 0.57 ± 0.045* |
| Thymus (%) | 0.06 ± 0.012 | 0.07 ± 0.021 | 0.05 ± 0.018 | 0.06 ± 0.017 |
| Heart (%) | 0.26 ± 0.029 | 0.25 ± 0.034 | 0.25 ± 0.026 | 0.24 ± 0.019 |
| Ovaries (%) | 0.03 ± 0.004 | 0.03 ± 0.006 | 0.03 ± 0.004 | 0.03 ± 0.005 |

[a]Values are presented as means ± SD.
*, **Significant difference at $p < 0.05$ and $p < 0.01$ levels, respectively, when compared with the control group.

90 mg/kg group were significantly higher than control group. The weights of liver in the 30 mg/kg group were also significantly increased in comparison to the control group (Table 5).

***Serum biochemistry.*** Table 6 shows the results of serum biochemistry tests obtained in the current study from pregnant dams. Significant increases in CHO and TG and significant decreases in BUN and GLU were observed in the 30 mg/kg group in comparison with the control group. The 90 mg/kg group showed significant increases in AST, ALT, CHO, TG, ALP and BIL and significant decreases in BUN, GLU, ALB and TP when compared with the control group.

***Caesarean section.*** Table 7 summarizes the reproductive findings for the pregnant rats treated with 1,3-DCP on days 6 through 19 of pregnancy. The overall

**Table 6.** Serum biochemical findings of pregnant rats treated with 1,3-dichloro-2-propanol during gestational days 6 through 19

| Items | 1,3-Dichloro-2-propanol (mg/kg/day) | | | |
|---|---|---|---|---|
| | 0 | 10 | 30 | 90 |
| AST (IU/l) | 92.2 ± 19.08[a] | 117.3 ± 18.04 | 201.3 ± 81.90 | 448.7 ± 187.49** |
| ALT (IU/l) | 39.8 ± 8.96 | 50.1 ± 10.25 | 124.4 ± 60.75 | 363.8 ± 143.77** |
| CHO (mg/dl) | 69.4 ± 9.82 | 77.8 ± 6.32 | 104.4 ± 32.35** | 203.5 ± 28.16** |
| TG (mg/dl) | 25.9 ± 6.45 | 29.1 ± 3.94 | 50.3 ± 19.00** | 57.8 ± 10.10** |
| BUN (mg/dl) | 21.9 ± 3.72 | 19.4 ± 0.92 | 17.2 ± 1.39** | 18.5 ± 3.89* |
| ALP (IU/l) | 257.0 ± 30.36 | 239.40 ± 32.63 | 247.11 ± 30.78 | 315.20 ± 61.30* |
| CRE (mg/dl) | 0.7 ± 0.063 | 0.7 ± 0.027 | 0.7 ± 0.039 | 0.7 ± 0.034 |
| GLU (mg/dl) | 131.2 ± 8.94 | 123.8 ± 10.43 | 117.4 ± 11.00* | 104.4 ± 7.24** |
| CA (mg/dl) | 7.9 ± 0.55 | 8.2 ± 0.57 | 8.2 ± 0.71 | 8.3 ± 0.70 |
| BIL (mg/dl) | 0.1 ± 0.020 | 0.1 ± 0.008 | 0.1 ± 0.047 | 0.2 ± 0.045** |
| ALB (g/dl) | 3.7 ± 0.22 | 3.8 ± 0.13 | 3.7 ± 0.18 | 3.5 ± 0.11** |
| TP (g/dl) | 6.0 ± 0.20 | 6.1 ± 0.10 | 6.0 ± 0.15 | 5.7 ± 0.16** |

[a]Values are presented as means ± SD.
*, **Significant difference at $p < 0.05$ and $p < 0.01$ levels, respectively, when compared with the control group.
AST, aspartate aminotransferase; ALT, alanine aminotransferase; CHO, cholesterol; TG, triglyceride; BUN, blood urea nitrogen; ALP, alkaline phosphatase; CRE, creatinine; GLU, glucose; CA, calcium; BIL, bilirubin; ALB, albumin; TP, total protein.

**Table 7.** Caesarean section data of pregnant rats treated with 1,3-dichloro-2-propanol during gestational days 6 through 19

| Items | 1,3-Dichloro-2-propanol (mg/kg/day) | | | |
|---|---|---|---|---|
| | 0 | 10 | 30 | 90 |
| Number of mated females | 10 | 10 | 10 | 10 |
| Number of pregnant females | 8 | 8 | 9 | 10 |
| Number of females totally resorbed | 0 | 0 | 0 | 0 |
| Corpora lutea | 13.3 ± 3.01[a] | 14.1 ± 2.23 | 14.6 ± 2.24 | 15.0 ± 1.94 |
| Implantation sites | 11.6 ± 3.89 | 12.1 ± 3.18 | 12.9 ± 1.90 | 14.0 ± 1.76 |
| Pre-implantation loss (%)[b] | 14.6 ± 14.19 | 14.5 ± 14.72 | 11.1 ± 9.14 | 6.2 ± 9.11 |
| Fetal deaths | | | | |
| Resorption: Early | 0.5 ± 0.76 | 0.8 ± 0.71 | 0.3 ± 0.50 | 0.6 ± 1.26 |
| Late | 0 | 0 | 0 | 0 |
| Dead fetuses | 0 | 0 | 0 | 0 |
| Post-implantation loss (%)[c] | 3.6 ± 5.45 | 5.8 ± 5.31 | 2.5 ± 3.76 | 4.3 ± 9.65 |
| Litter size | 11.1 ± 3.64 | 10.4 ± 2.83 | 12.6 ± 1.88 | 13.4 ± 2.17 |
| Male/female | 47/42 | 46/37 | 61/52 | 61/73 |
| Sex ratio | 1.12 | 1.24 | 1.17 | 0.84 |
| Fetal weight (g): Male | 4.4 ± 0.12 | 4.2 ± 0.13 | 4.2 ± 0.33 | 4.1 ± 0.32* |
| Female | 4.2 ± 0.14 | 4.1 ± 0.20 | 4.0 ± 0.34 | 3.9 ± 0.37** |
| Placental weight (g) | 0.5 ± 0.08 | 0.5 ± 0.07 | 0.6 ± 0.08 | 0.6 ± 0.12 |

[a]Values are presented as means ± SD.
[b]Pre-implantation loss (%) = [(No. of corpora lutea - No. of implantation sites)/No. of corpora lutea] × 100.
[c]Post-implantation loss (%) = [(No. of implantation sites - No. of live embryos)/No. of implantation sites] × 100.
*, ** Significant difference at $P < 0.05$ and $P < 0.01$ levels, respectively, when compared with the control group.

pregnancy rates were comparable across all dosage groups, ranging from 80~100%. No totally resorbed litters were found in any group. There were no treatment-related effects on the number of corpora lutea, implantations, pre- and post-implantation loss rates, fetal deaths, litter size, sex ratio of the live fetuses, fetal weight, and placental weight in any of the treated animals.

## DISCUSSION

The present study was carried out to evaluate the potential maternal toxicity of 1,3-DCP administered by oral gavage to Sprague-Dawley rats at dose levels of 0, 10, 30, and 90 mg/kg per day on days 6 through 19 of pregnancy. The results of this study showed that a 14-day oral dose of 1,3-DCP to rats during pregnancy resulted in various adverse effects on clinical sign, body weight, food consumption, organ weight, and serum biochemistry at dose levels of ≥ 30 mg/kg/day.

Treatment-related clinical signs were observed in the 30 and 90 mg/kg groups, which were evidenced by the dose-dependent increase in the incidence and severity of piloerection, abnormal gait, decreased locomotor activity, a reddish tear from the eyes, loss of fur, and nasal discharge. These findings were clear indications of stress and/or malnutrition induced by the treatment of 1,3-DCP. This interpretation is supported by increased relative adrenal weight associated with decreased food intake and suppressed body weight gain. It is thought that these clinical signs can be attributed to the irritation of gastrointestinal (GI) tract and systemic toxic effects of 1,3-DCP (Smyth *et al.*, 1962; RTECS, 2000).

Significant maternal toxicity of 1,3-DCP was observed in the 30 and 90 mg/kg groups, which was evidenced by the decrease in body weight, body weight gain, corrected body weight, and food consumption, the increase in adrenal gland, and liver weights, and serum biochemical changes.

The dose-dependent decrease of body weight gain observed in the 90 mg/kg group was attributed to the exposure to the test chemical, which is consistent with the decreased food consumption observed in the group during the study period. This is a clear indication of the general toxicity induced by 1,3-DCP, which suggests that oral exposure to this chemical causes mild anorexia, followed by the suppression of body weight gain in rats. According to the report of Jersey *et al.* (1991), repeated oral administration of 1,3-DCP caused a significant decrease in body weight gain and food consumption when it was administered to rats by gavage at 10 mg/kg/day for 13 weeks. Recently, we have also demonstrated that 13-week repeated inhalation exposure of rats to 1,3-DCP resulted in suppressed body weight gain in rats (Kim *et al.*, 2007).

It is well known that body weight and organ weight are sensitive indicators of potentially toxic chemicals in general toxicity studies (Andersen *et al.*, 1999; Kim *et al.*, 2004). As described above, oral administration of rats to 1,3-DCP caused a significant decrease in body weight gain in the 90 mg/kg group. The suppressed body weight affected the relative weights of kidneys in the high-dose group. The weight change in the kidneys is of uncertain toxicological significance because there was no corresponding serum biochemical change that accompanied the difference in organ weight. On the contrary, the increased absolute and relative liver weights observed in the 30 and 90 mg/kg groups and the increased relative adrenal glands weight found in the 90 mg/kg group indicate that these findings are closely correlated to the treatment of 1,3-DCP, since the relevant serum biochemical changes were detected in the groups.

Serum aminotranferase activities have long been considered to be sensitive indicators of hepatic injury (Molander *et al.*, 1955). Injury to the hepatocytes alters their transport function and membrane permeability, leading to leakage of enzymes from the cells (Zimmerman and Seeff, 1970). Therefore, the marked release of AST and ALT into the circulation indicates severe damage to hepatic tissue membranes during intoxication. The significant increase of serum AST and ALT activities observed in the 90 mg/kg group was considered to be due to 1,3-DCP treatment, because the increment was remarkable (386~814%) and showed a clear-cut dose-response relationship. The hepatotoxic effects induced by 1,3-DCP administration were also confirmed by other serum biochemical parameters including increased CHO and TG and decreased GLU in the 30 mg/kg group and increased CHO, TG, ALP, and BIL and decreased GLU, ALB, and TP in the 90 mg/kg group. The significant decrease of BUN observed in the 30 and 90 mg/kg groups was of no toxicological significance because renal dysfunction is associated with increased serum levels of BUN and CRE (Lesko and Mitchell, 1997).

The potential toxicity of 1,3-DCP has been studied extensively over the past several decades using both short- and long-term animal tests. The acute oral $LD_{50}$ of 1,3-DCP ranged from 100 mg/kg to 400 mg/kg in rats, and ranged from 25 mg/kg to 125 mg/kg in mice, depending on the purity of 1,3-DCP and the rodent sex and strain used (RTECS, 2000; HSDB, 2002). Limited data have been reported on the potential repeated dose toxicity of 1,3-DCP. According to the report of Jersey *et al.* (1991), 13 weeks of oral repeated doses of 1,3-DCP to rats cause decreased body weight gain and feed consumption, altered hematological parameters, increased liver and kidney weights, alterations in serum chemistry and urinary parameters, gross pathological changes in the stomach, and histopathological changes in the stomach, kidney, liver and nasal tissue at 100 mg/kg/day; it also caused increased liver weights and histopathological changes in the stomach, kidneys and liver at 10 mg/kg/day. In a more recent study (Lym *et al.*, 2003), Sprague-Dawley rats given 1,3-DCP at 15, 30, or 60 mg/kg/day daily by gavage for 13 weeks exhibited dose-dependent increases in liver and kidney weights. In males only, an increase in albumin and dose-dependent decreases in WBC, MCV and MCH were observed. In females, RBC, hemoglobin, hematocrit, MCH, MCHC, and WBC were slightly decreased and platelets and total cholesterol were increased. We have also reported that the whole-body inhalation exposure to 1,3-DCP for 13 weeks resulted in various adverse effects on body weight, liver, kidney and blood cells at concentrations > 5 ppm in rats (Kim *et al.*, 2007). The results reported by the above researchers and the present study clearly showed that 1,3-DCP is a hepatotoxic agent in both growing and pregnant rats.

In conclusion, the 14-day repeated oral dose of pregnant rats to 1,3-DCP resulted in decreases in the food consumption and GLU and increases in the liver weight, CHO and TG at 30 mg/kg/day; decreases in the body weight gain, food consumption, GLU, ALB, and TP and increases in the weights of liver and adrenal glands, AST, ALT, CHO, TG, ALP and BIL at 90 mg/kg/day. The target organ was determined to be the liver in pregnant rats. The no-observed-adverse-effect level was considered to be 10 mg/kg/day in rats.

## ACKNOWLEDGEMENTS

This work was supported by the Grant of the Korean Ministry of Education, Science and Technology (The Regional Core Research Program/Biohousing Research Institute).
